# Association of Psoas Muscle Mass at Intensive Care Unit Admission With Physical Function and Post-discharge Destination in Survivors of Critical Illness

**DOI:** 10.7759/cureus.59609

**Published:** 2024-05-03

**Authors:** Tanaka Kohei, Daisuke Takamura, Shota Nonaka, Tomoki Yamada

**Affiliations:** 1 Department of Rehabilitation Medicine, Osaka Police Hospital, Osaka, JPN; 2 Department of Rehabilitation Science, Graduate School of Health Science, Kobe University, Kobe, JPN; 3 Department of Rehabilitation, Kobe City Medical Center General Hospital, Kobe, JPN; 4 Department of Radiology Technology, Osaka Police Hospital, Osaka, JPN; 5 Emergency Critical Care Medical Center, Osaka Police Hospital, Osaka, JPN

**Keywords:** skeletal muscle mass, psoas muscles, physical and rehabilitation medicine, functional status assessment, intensive care units

## Abstract

Objective: Survivors of critical illness may have physical impairments, known as post-intensive care syndrome (PICS). Early screening for the risk of PICS is recommended to prevent PICS. Skeletal muscle mass is a clinically important indicator associated with various outcomes. This study aimed to examine the association of psoas muscle mass at intensive care unit (ICU) admission with the destination and physical function at hospital discharge.

Methods: In this single-center retrospective cohort study, we reviewed the medical records of adult patients who had required emergency ICU admission and who had been intubated and mechanically ventilated. Psoas major muscle was measured as an indicator of skeletal muscle mass from abdominal computed tomography images at ICU admission. Physical function was assessed using the functional status score for the ICU and ICU mobility scale at hospital discharge. Multinomial logistic and multivariable linear regression were used to analyze the associations of the psoas muscle mass with the discharge destination and physical function at discharge.

Results: We enrolled 124 patients (79 men and 45 women) with a median (interquartile range) age of 72.0 (62.0-80.0) years; 39 (31.5%) were discharged to home, 50 (40.3%) were transferred to rehabilitation wards, and 35 (28.2%) were transferred to long-term care settings. The psoas muscle area and volume were 16.9 (11.3-20.6) cm^2^ and 228.3 (180.2-282.0) cm^3^ in home discharge patients, 17.5 (11.5-21.5) cm^2^ and 248.4 (162.0-311.4) cm^3^ in rehabilitation ward patients, and 15.9 (10.3-19.5) cm^2^ and 184.0 (137.0-251.1) cm^3^ in long-term care patients. The areas and volumes of the psoas muscle were not significantly different in the three groups. Furthermore, psoas muscle mass was not significantly associated with the discharge destination and physical function.

Conclusions: Discharge destination and physical function at hospital discharge were not significantly associated with psoas muscle mass at ICU admission.

## Introduction

Survivors of critical illness may experience difficulty returning to their pre-hospitalization lifestyles due to impairments in physical, cognitive, or mental health status, known as post-intensive care syndrome (PICS) [1]. PICS is a long-term problem that persists several years after hospital discharge [2]. Many patients suffer difficulty performing basic activities of daily living (ADL), such as standing up and walking [3,4], which results in a decline in health-related quality of life [5,6]. Acute skeletal muscle loss and muscle weakness are observed during critical care [7]. Up to two-thirds of patients are diagnosed with intensive care unit-acquired weakness (ICU-AW) [8]. Rapidly progressive muscle atrophy and ICU-AW are major contributing factors for PICS. Physical therapy including early mobilization is considered an essential component of the multidisciplinary management of patients in the intensive care unit (ICU) [9,10]. Early physical therapy provides significant improvements in short-term physical-related outcomes [11]. Screening the risk factors for PICS as early as possible in the ICU is recommended to prevent PICS [10]. However, measurable parameters early in ICU admission have not been clearly validated to assess the risk of PICS. In various settings, skeletal muscle mass has been associated with various outcomes, including physical function [12-14]. One study revealed that the skeletal muscle mass of patients with critical illness is associated with mortality and ventilation-free days [15-17]. At emergency admission, computed tomography (CT) images are taken frequently, and abdominal CT images could be used to measure the psoas major muscle. Therefore, measuring the psoas muscle mass in patients who had abdominal CT scans is reasonable in that it does not require additional testing. In this study, we hypothesized that skeletal muscle mass, as measured by the psoas major muscle, is also a predictor of physical function in critically ill patients. Although no international consensus exists for assessing physical impairment after critical care, the availability of home discharge and the ability of basic ADL are seemingly important indicators for patients. Moreover, predicting the discharge destination and physical function after critical care may enable medical staff to consider rehabilitation goals and interventions for these patients prior to the event. Therefore, this study aimed to examine the association of psoas muscle mass at ICU admission and the destination and physical function at hospital discharge.

## Materials and methods

Study population

This single-center retrospective cohort study was conducted at Osaka Police Hospital (Osaka, Japan). Medical records of patients from April 2019 to March 2021 were reviewed accordingly. This study included adult patients (20 years and over) who had required emergency admission to the Osaka Police Hospital and who had been intubated and mechanically ventilated for more than two days. Patients were excluded if they had died during hospitalization; had experienced acute or chronic neuromuscular disease, fracture, or limb amputation; or had withdrawn from treatment. This study was approved by the Research Ethics Committee of Osaka Police Hospital (approval number: 1627). An opt-out method was used on the hospital website to provide participants with the opportunity to decline participation in the study.

Outcome measures

**Figure 1:**
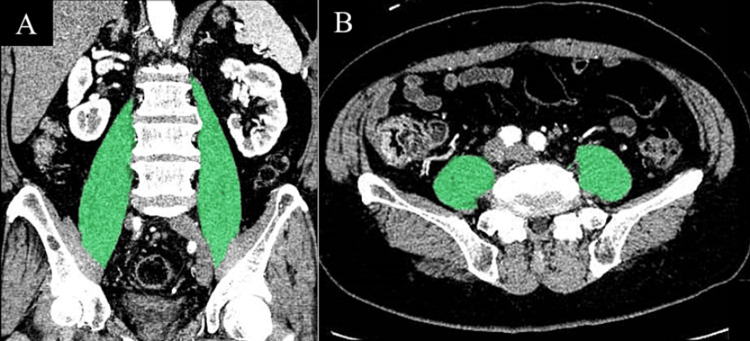
Measurements of the psoas muscle area and volume. (A) The psoas muscle volume was measured at the vertebral level from the diaphragm to the pubic symphysis using automated analysis with Synapse Vincent (Fujifilm, Japan). (B) The psoas muscle area was measured at the middle level of the third lumbar vertebra using a single axial CT scan image.

The primary outcome was the association between the skeletal muscle mass at ICU admission and the discharge destination. The area and volume of the psoas major muscle were used as indicators of skeletal muscle mass. The psoas muscle mass was measured using abdominal computed tomography (CT) images at ICU admission using automated analysis with Synapse Vincent (Fujifilm, Japan). The volume of the psoas muscle was measured at the vertebral level from the diaphragm to the pubic symphysis (Figure 1A). The area of the psoas muscle was measured at the third lumbar vertebra with the transverse process vertebrae visible (Figure 1B). The participants were categorized into three groups according to their discharge destinations: (1) home, (2) rehabilitation ward, and (3) long-term care setting. In this context, post-acute care hospitals and inpatient rehabilitation hospitals were grouped together as “rehabilitation wards.” Nursing homes, hospitals with long-term care beds, and other places were grouped together as “long-term care.”

The secondary outcome was the association between the skeletal muscle mass at ICU admission and physical function at hospital discharge. The functional status score for the ICU (FSS-ICU) and the ICU mobility scale (IMS) were used for the assessment of physical function. The FSS-ICU is a mobility assessment scale based on the difference in level of assistance with daily activities [18,19]. The FSS-ICU is evaluated in five activities: rolling, transfer from supine to sitting position, sitting at the edge of the bed, transfer from the sitting to the standing position, and ambulation. Each activity is rated with an 8-point ordinal scale with scores ranging from 0 (not able) to 7 (fully independent), with a maximum cumulative score of 35. The maximum level of mobility was assessed by IMS. The IMS assesses the maximum level of mobility with an 11-point rating scale, with scores ranging from 0 (lying and passive exercises in bed) to 10 (independent ambulation) [20]. The FSS-ICU and IMS scores were assessed by physical therapists at hospital discharge.

Other measurements

The disease severity was assessed at ICU admission by using the sequential organ failure assessment (SOFA) score, a scoring system used to quantify organ dysfunction [21]. Physical condition before ICU admission was assessed using the clinical frailty scale (CFS) on a 9-point rating scale with scores from 1 (very fit) to 9 (terminally ill) [22,23]. The independence level of daily activities prior to ICU admission was assessed by using the Katz index of independence in ADL (Katz ADL) scale. The original scale was based on seven categories for each level of care dependency and was revised in 1973. The revised scale assesses six ADLs, including bathing, dressing, toileting, transferring, continence, and feeding, with a binary rating scale (dependent or independent). It is classified into categories A to G [24]. The CFS and Katz ADL scores were assessed retrospectively based on the information provided by patients themselves and their families as accumulated in the medical records.

Statistical analysis

Data were presented using median and interquartile range (IQR). Kruskal-Wallis tests were used to compare clinical variables between the three groups. The association of skeletal muscle mass with discharge destination was analyzed by multinomial logistic regression analysis. Multivariable linear regression analysis was used to analyze the association between skeletal muscle mass and each of FSS-ICU and IMS at hospital discharge. The area and volume of the psoas muscles were used respectively as indices of skeletal muscle mass. Covariates for adjusting bias were as follows: age, sex, body mass index (BMI), CFS, SOFA score, and duration of mechanical ventilation. Multicollinearity was assessed using the variance inflation factor. A variance inflation factor value from 1 to 10 was considered the absence of multicollinearity. The sample size for multinomial logistic regression analysis was calculated with five events per variable, after referring to a previous study [25]. In this study, we planned three categorizations and seven explanatory variables, and the resultant sample size was set at 105 participants (35/group). All data were analyzed using the statistical software Statistical Package for Social Sciences (SPSS) for Windows, version 21.0 (IBM Corp., Armonk, NY). Statistical significance was set at p < 0.05.

## Results

This study enrolled 124 patients (79 (63.7%) men and 45 (36.3%) women) with a median age (IQR) of 72.0 (62.0-80.0) years. The patients’ clinical characteristics are summarized in Table 1. An analysis of the data revealed that 39 patients (31.5%) had been discharged to home, 50 (40.3%) had been transferred to a rehabilitation ward, and 35 (28.2%) had been transferred to long-term care settings. The psoas muscle area was 16.9 (11.3-20.6) cm^2^ in home discharge patients, 17.5 (11.5-21.5) cm^2^ in rehabilitation ward patients, and 15.9 (10.3-19.5) cm^2^ in long-term care patients; the psoas muscle volume was 228.3 (180.2-282.0) cm^3^, 248.4 (162.0-311.4) cm^3^, and 184.0 (137.0-251.1) cm^3^, respectively. The areas and volumes of the psoas major muscle were not significantly different in the three groups. Patients in the long-term care group were significantly older than the patients in the home discharge (p < 0.01) and rehabilitation ward groups (p = 0.03), and their CFS (p < 0.01, each group) and Katz ADL (p < 0.01, each group) scores were worse. Patients in the long-term care group had significantly lower BMI than other group patients (p < 0.01, each group). The duration of mechanical ventilation in the home-discharged group was significantly shorter (p = 0.04, p < 0.01, respectively) and their SOFA score at ICU admission was significantly lower (p < 0.01, each group). The FSS-ICU and IMS scores at hospital discharge were significantly higher in home discharge patients than those of rehabilitation group and long-term care group (p < 0.01, in all comparisons). Furthermore, the FSS-ICU scores were higher in the rehabilitation group than in the long-term care group (p < 0.01).

**Table 1:** Baseline characteristics of participants and between-group comparison according to the presence of dysphagia. Data are presented as median (IQR) and N (%). Statistical significance was set at p < 0.05. The p-value in this table indicates the results of the Kruskal-Wallis test. ARDS, acute respiratory distress syndrome; BMI, body mass index; FSS-ICU, functional status score for the ICU; IMS, ICU mobility scale; SOFA, sequential organ failure assessment; Katz ADL, Katz index of independence in activities of daily living; PMV, psoas muscle volume.

	Total (n=124)		Home (n=39)		Rehabilitation ward (n=50)		Long-term care (n=35)		P-value
Age (years)	72.0	(62.0-80.0)	69.0	(58.5-74.0)	72.0	(54.3-80.5)	78.0	(71.5-82.5)	0.001
Sex, n (%)									0.633
-Male	79	(63.7)	26	(66.7)	33	(66.0)	20	(57.1)	
-Female	45	(36.3)	13	(33.3)	17	(34.0)	15	(42.9)	
BMI (kg/m^2^)	22.7	(19.9-24.9)	22.9	(21.5-25.2)	24.1	(20.0-25.7)	20.1	(18.3-22.8)	0.001
Primary diagnosis, n (%)									
-Sepsis	51	(41.1)	16	(41.0)	24	(48.0)	11	(31.4)	
-ARDS	19	(15.3)	7	(17.9)	4	(8.0)	8	(22.9)	
-Hemorrhagic shock	12	(9.7)	5	(12.8)	6	(12.0)	1	(2.9)	
-Trauma	12	(9.7)	3	(7.7)	9	(18.0)	0	(0)	
-Acute heart failure	4	(3.2)	2	(5.1)	1	(2.0)	1	(2.9)	
-Burns	4	(3.2)	0	(0)	2	(4.0)	2	(5.7)	
-Others	22	17.7)	6	(15.4)	4	(8.0)	12	(34.3)	
SOFA score	6.0	(3.0-9.0)	3.0	(2.0-7.0)	7.0	(4.0-9.0)	7.0	(5.5-10.0)	0.001
Clinical frailty scale	2.5	(2.0-5.0)	2.0	(2.0-3.0)	2.0	(2.0-3.8)	6.0	(2.5-6.0)	<0.001
Katz ADL score	7.0	(7.0-7.0)	7.0	(7.0-7.0)	7.0	(7.0-7.0)	5.0	(1.0-7.0)	<0.001
Duration of mechanical ventilation (days)	6.0	(4.0-11.0)	5.0	(2.5-6.0)	7.0	(4.0-12.0)	10.0	(6.0-12.0)	<0.001
Length of ICU stay (days)	9.0	(5.0-14.0)	8.0	(4.0-9.5)	8.5	(5.0-15.8)	12.0	(8.5-15.5)	0.005
Length of hospital stay (days)	30.5	(21.0-43.3)	26.0	(18.5-39.5)	31.0	(22.0-45.3)	36.0	(24.0-46.0)	0.102
FSS-ICU	30.5	(18.5-35.0)	35.0	(33.0-35.0)	32.5	(22.5-35.0)	14.0	(4.5-24.0)	<0.001
IMS	9.0	(5.8-10.0)	10.0	(9.0-10.0)	9.0	(8.0-10.0)	5.0	(3.5-8.0)	<0.001
Psoas muscle area (cm^2^)	17.0	(11.0-20.6)	16.9	(11.3-20.6)	17.5	(11.5-21.5)	15.9	(10.3-19.5)	0.572
Psoas muscle volume (cm^3^)	206.7	(157.8-295.2)	228.3	(180.2-282.0)	248.4	(162.0-311.4)	184.0	(137.0-251.1)	0.132

Multinomial logistic regression was used to analyze the association between skeletal muscle mass and discharge destination. Table 2 shows the odds ratios based on patients discharged home as representative. The psoas major muscle area (home vs. rehabilitation, 95% CI: 0.937-1.196; home vs. long-term care, 95% CI: 0.937-1.313) and volume (home vs. rehabilitation, 95% CI: 0.996-1.011; home vs. long-term care, 95% CI: 0.996-1.019) were not significantly associated with discharge destination. Although patients from rehabilitation wards were used as a reference, multinomial logistic regression analysis also revealed no significant associations of the psoas major muscle area (rehabilitation vs. long-term care, 95% CI: 0.991-1.018) and volume (rehabilitation vs. long-term care, 95% CI: 0.860-1.291) with discharge destination.

**Table 2:** Multinomial logistic regression analysis for the association of skeletal muscle mass and discharge destination. The table shows the odds ratios for discharge destination based on patients discharged home. Model 1 used area and Model 2 used volume of psoas major muscle in analysis. Each model analysis was adjusted by covariate factors as follows: age, sex, body mass index, clinical frailty scale, SOFA score, and duration of mechanical ventilation. Statistical significance was set at p < 0.05. SOFA, sequential organ failure assessment.

	Odds ratio	P-value	95% CI	
			Lower	Upper
Model 1				
-Rehabilitation ward	1.059	0.357	0.937	1.196
-Long-term care	1.109	0.228	0.937	1.313
Model 2				
-Rehabilitation ward	1.004	0.326	0.996	1.011
-Long-term care	1.008	0.177	0.996	1.019

Similarly, multivariable linear regression analysis showed no significant relationship between skeletal muscle mass and the FSS-ICU and IMS scores at hospital discharge (Table 3).

**Table 3:** Multivariable linear regression analysis for the association of skeletal muscle mass and physical function at hospital discharge. Each of the objective variables was analyzed with the area and volume of the psoas major muscle respectively, and were adjusted by covariate factors as follows: age, sex, body mass index, clinical frailty scale, SOFA score, and duration of mechanical ventilation. Statistical significance was set at p < 0.05. IMS, ICU mobility scale; SOFA, sequential organ failure assessment.

Object variables	Clinical variables	Coefficient		P-value	95% CI	
		B	β		Lower	Upper
FSS-ICU	Area of psoas major muscle	0.024	0.014	0.897	-0.346	0.395
	Volume of psoas major muscle	0.005	0.046	0.665	-0.018	0.029
IMS	Area of psoas major muscle	-0.058	-0.137	0.206	-0.149	0.032
	Volume of psoas major muscle	-0.002	-0.060	0.592	-0.008	0.004

## Discussion

Our results revealed that psoas major muscle mass at ICU admission was not significantly associated with the discharge destination and physical function at hospital discharge. Compared with patients discharged to home, those who had been transferred to other places had longer durations of mechanical ventilation, higher severity of illness, and poorer physical function before ICU admission. CT scan is commonly obtained for critically ill patients requiring mechanical ventilation on emergency admission to search for the causes. Some patients may have abdominal CT scans; thus, assessing the psoas muscle mass has an advantage as it can be determined without additional testing. However, it would not have been an appropriate indicator for predicting physical conditions at hospital discharge.

Skeletal muscle plays an essential role in maintaining posture and joint mobility and is strongly associated with ADL. Skeletal muscle mass is positively correlated with muscle strength and physical function [26]. A decrease in muscle volume can lead to limitations in ADL and a decline in quality of life [27,28]. Low muscle volume predicts poor clinical outcomes in various diseases [12-14]. One study reported an association between skeletal muscle mass and mortality of patients with critical illness [15-17]. Conversely, our results showed that discharge destination and physical function at hospital discharge were not significantly associated with psoas muscle mass.

Rapid skeletal muscle loss occurs during intensive care, and this begins immediately after ICU admission [29]. In the acute phase of severe disease, systemic inflammatory condition increases inflammatory cytokines and acute-phase proteins and decreases the synthesis of visceral proteins [30]. The skeletal muscle is the source of endogenous energy during the acute phase of the disease. The synthesis of hepatic gluconeogenesis is upregulated and skeletal muscle catabolism is rapidly accelerated during critical care. The degree of skeletal muscle loss during intensive care is dependent on disease severity and the presence of organ failure [31]. In this study, longitudinal skeletal muscle measurements were not available; thus, the degree of muscle alteration in the patients was unclear. The amount of muscle loss would have been less in patients who were home-discharged since their severity of illness on admission was significantly lower than that of patients who were transferred. Although a study showed that low skeletal muscle mass at ICU admission predicts skeletal muscle weakness at ICU discharge [32], it would have been difficult to use skeletal muscle mass, which changes significantly after ICU admission, to predict physical function at hospital discharge. Furthermore, muscle weakness in ICU survivors results from heterogeneous muscle pathophysiology with variable combinations of muscle atrophy and impaired voluntary contractile capacity [33]. Muscle alterations, such as muscle power and muscle quality, developed after ICU admission are predictors of physical function at hospital discharge in patients surviving critical illness [34]. Predicting physical function after intensive care may be difficult based on skeletal muscle mass assessed at a single point alone, and therefore the skeletal muscle may have to be assessed comprehensively and longitudinally.

This study had some limitations. The area and volume of the psoas major muscle were used as indicators of whole-body skeletal muscle mass. the psoas major muscle is correlated with total body skeletal muscle mass [35], and measurement of psoas major muscle mass is an excellent screening method in clinical practice since it can be analyzed from existing CT images. However, the psoas major muscle actually has a small volume in terms of whole-body skeletal muscle mass. The dual-energy X-ray absorptiometry or bioelectrical impedance analysis may be desirable to measure whole-body skeletal muscle mass. Furthermore, discharge destination and physical function measured by FSS-ICU and IMS were used as indicators of physical impairment in PICS, however, various phenotypes of physical impairment occur after intensive care, such as muscle weakness, impaired mobility, and poor endurance, which were not considered in the present study. The association between skeletal muscle mass and various indicators of physical function may require investigation. Further studies are thus needed to investigate the validated methods for screening the risk of PICS development as early as ICU admission.

## Conclusions

Skeletal muscle mass has been reported to be associated with clinical outcomes in various conditions. However, the discharge destination and physical function at hospital discharge of survivors of critical illness were not significantly associated with the psoas major muscle mass at ICU admission. Physical function after intensive care may be affected by various factors during treatment. Comprehensive and repetitive muscle assessments may be necessary to screen for PICS risk and prevent its onset.
